# Effects of Prepartum Flaxseed and *Hermetia illucens* Oil Supplementation on Fatty Acid Profiles Across the Maternal–Placental–Offspring Axis in Prolific Ewes

**DOI:** 10.3390/ijms27219740

**Published:** 2026-10-30

**Authors:** Kamila Lewandowska, Natalia Pachura-Hanusek, Piotr Kuropka, Antoni Szumny, Robert Kupczyński

**Affiliations:** 1 Department of Environmental Hygiene and Animal Welfare, Faculty of Biology and Animal Science, Wrocław University of Environmental and Life Sciences, Chełmońskiego St. 38c, 51-630 Wrocław, Poland; natalia.pachura@upwr.edu.pl; 2 Department of Biostructure and Animal Physiology, Faculty of Veterinary Medicine, Wrocław University of Environmental and Life Sciences, Norwida St. 31, 50-375 Wrocław, Poland; piotr.kuropka@upwr.edu.pl; 3 Department of Food Chemistry and Biocatalysis, Faculty of Biotechnology and Food Science, Wrocław University of Environmental and Life Sciences, Norwida St. 27, 50-375 Wrocław, Poland; antoni.szumny@upwr.edu.pl

**Keywords:** flaxseed oil, black soldier fly larvae oil, fatty acid profile, placenta, amniotic fluid, lambs, maternal–offspring axis

## Abstract

Maternal dietary lipids may influence fatty acid availability across the maternal–placental–offspring axis. This study evaluated the effects of prepartum supplementation with flaxseed oil (FO), black soldier fly larvae (BSFL; *Hermetia illucens*) oil, or their 2:1 blend (Mix) in 32 pregnant Olkuska ewes. Ewes in the supplemented groups received 30 g of oil daily for 21 days during late gestation. Fatty acid profiles were determined in maternal serum, placental tissue, amniotic fluid, and lamb serum, together with placental histology and early lamb performance. Maternal serum exhibited the most pronounced treatment- and time-related changes, including increased C14:0 in the BSFL and Mix groups, whereas placental tissue and amniotic fluid showed more limited, compartment-specific responses. In lamb serum collected after colostrum intake, postnatal age was strongly associated with fatty acid composition, whereas nominal treatment-related differences did not withstand FDR adjustment. Placental architecture remained preserved across treatments, without inflammatory, degenerative, or vascular lesions. Lamb growth performance did not differ significantly among treatments. Overall, maternal lipid supplementation was associated with compartment-specific differences in relative fatty acid composition. Because placental fatty acid transport was not directly assessed, these findings do not demonstrate direct maternal-to-offspring fatty acid transfer.

## 1. Introduction

Late gestation is a critical period for fetal development in sheep, and a time of rapid fetal growth and great increases in the requirements for nutrients and oxygen [1,2,3,4]. Highly prolific sheep breeds, such as the Olkuska, represent valuable animal models for studying multiple pregnancy. The high prolificacy of the Olkuska breed has been associated with genetic variation in the BMP15 gene, making this breed particularly relevant for studying physiological adaptations to multifetal gestation [5]. Increased maternal metabolic demands during twin and triplet gestations, particularly in late pregnancy, highlight the potential benefits of dietary lipid supplementation for supporting energy supply and fatty acid metabolism. Compared with singleton pregnancies, multifetal gestations are associated with greater maternal energy requirements and may intensify lipid mobilization, particularly when dietary energy intake is insufficient [6,7]. Furthermore, studies comparing singleton and twin pregnancies in sheep have demonstrated differences in placental morphology and nutrient transport-related parameters, highlighting the importance of placental function in supporting fetal development during multifetal gestation [8]. The placenta is the key interface between the maternal and fetal compartments and plays active roles in nutrient and gas exchange, placental metabolism, endocrine signaling and modulation of immune interactions at the maternal–fetal interface [9,10,11,12]. Ovine placentation is chorioallantoic, villous, cotyledonary and synepitheliochorial [13]. Placentomes are formed by the fusion of maternal caruncles and fetal cotyledons and are the primary sites of hemotrophic exchange between the maternal and fetal circulations [14,15,16]. Thus, adequate uteroplacental blood flow, placental vascular development, and preservation of placental structural integrity are essential to maintain nutrient and oxygen delivery and to support the increasing metabolic demands of the fetus during late gestation [17,18,19].

In addition to its transport function, the morphological condition of the placenta provides important information regarding the quality of the maternal–fetal interface. Histological evaluation enables assessment of the integrity of the villous epithelium and stromal organization as well as detection of inflammatory, degenerative and vascular alterations. Placental inflammation may be associated with epithelial damage, necrosis, inflammatory cell infiltration, edema and vascular lesions, potentially compromising materno-fetal exchange [13]. For this reason, evaluation of placental morphology may provide complementary information when investigating nutritional interventions that could influence maternal metabolism and placental homeostasis.

Fatty acids are important components of the maternal–placental–fetal axis, providing metabolic substrates for the developing fetus while also contributing to cell membrane structure and lipid signaling. Their transfer across the placenta is a regulated process rather than simple passive diffusion. Fatty acids taken up from the maternal circulation can be bound intracellularly, esterified, oxidized, or incorporated into placental lipid pools before becoming available to the fetus. Placental lipid metabolism may therefore influence both the amount and composition of fatty acids reaching the fetal compartment [20,21]. Marked differences in fatty acid composition between maternal serum, placental tissue, and fetal serum have been reported in sheep [22], while more recent studies demonstrate that placental fatty acid transport and lipid metabolism are responsive to maternal nutritional status [20,23].

Maternal dietary lipid supply may consequently influence fatty acid availability at several levels of the maternal–fetal system. In late-gestation ewes, dietary supplementation with linoleic acid (LA) has been shown to alter fatty acid profiles in maternal and fetal serum as well as placental tissue, together with changes in placental prostaglandin synthesis [24]. Supplementation with long-chain polyunsaturated fatty acids (LC-PUFAs) during late pregnancy has also been associated with changes in the fatty acid composition of maternal plasma, colostrum, milk, and offspring circulation [25,26]. However, fatty acid profiles measured in lambs after birth may already reflect the contribution of colostrum and milk intake. Examining maternal serum, placental tissue, fetal fluids, and offspring circulation within the same experimental model may therefore provide a more complete view of fatty acid distribution across the maternal–placental–offspring axis. In the present study, the term “maternal–placental–offspring axis” refers to the biological compartments examined within the same experimental model rather than a demonstrated pathway of fatty acid transfer.

The lipid sources used in the present study differed markedly in their fatty acid composition. Unlike conventional flaxseed oil, which is typically rich in α-linolenic acid, the flaxseed oil used in this experiment exhibited an atypical fatty acid profile. The flaxseed oil (FO) was characterized by a high proportion of linoleic acid (C18:2 n-6) and a low proportion of α-linolenic acid (C18:3 n-3, ALA), whereas black soldier fly larvae (BSFL; *Hermetia illucens*) oil contained a high proportion of lauric acid (C12:0). The selection of these two lipid sources was based on their contrasting fatty acid compositions and previous in vitro investigations of their effects on ruminal fermentation. The 2:1 flaxseed-to-BSFL oil ratio was adopted to reduce the contribution of lauric acid-rich insect oil while maintaining both lipid sources in the experimental formulation [27]. Insect-derived lipids are increasingly being considered as alternative dietary fat sources in animal nutrition, but their use in sheep has been studied only to a limited extent [28,29,30]. Recent in vivo work in lactating ewes has focused mainly on ruminal fermentation, animal performance, blood metabolites, and oxidative status [29]. By contrast, little is known about the effects of lauric acid-rich BSFL oil supplementation during pregnancy, particularly in relation to placental fatty acid transfer, fetal lipid utilization, and fatty acid profiles in placental tissue, amniotic fluid, and neonatal circulation.

Alongside the assessment of placental morphology, the presence and distribution of bacterial-like structures within placental tissue may provide complementary information on local tissue status. Because expelled placentas are exposed to the external environment, structures exhibiting bacterial morphology observed on the placental surface may partly reflect postpartum contamination and should therefore be interpreted with caution. In this context, observations from deeper placental compartments, considered together with histological evidence of inflammation or tissue damage, may provide additional insight into placental tissue integrity.

Thus, this study aimed to determine whether maternal supplementation with high-linoleic flaxseed oil, black soldier fly larvae oil, or their 2:1 blend (Mix) during the final 21 days of gestation influenced fatty acid profiles across the maternal–placental–offspring axis. Fatty acid composition was assessed in maternal serum, placental tissue, amniotic fluid, and lamb serum. We hypothesized that prepartum supplementation with lipid sources differing in fatty acid composition would induce source-specific changes in maternal fatty acid profiles, particularly in fatty acids associated with the supplemented oils, including linoleic acid (C18:2 n-6) and lauric acid (C12:0). Specifically, we expected flaxseed oil supplementation to increase the relative proportion of linoleic acid (C18:2 n-6), whereas BSFL oil supplementation was expected to increase the proportion of lauric acid (C12:0) in maternal serum. The mixed treatment was expected to produce an intermediate or combined response. We hypothesized that these responses would vary across biological compartments, with more pronounced changes in maternal serum than in placental tissue and amniotic fluid, potentially reflecting differences in lipid metabolism and regulation at the maternal–fetal interface. In offspring, we expected maternal dietary supplementation to influence selected circulating fatty acids, with responses potentially modified by postnatal nutritional and metabolic adaptations.

## 2. Results

### 2.1. Maternal Serum Fatty Acid Profile

Maternal serum fatty acid composition showed both time-dependent and treatment-specific responses to prepartum lipid supplementation (Table 1). A significant treatment × time interaction was observed for C14:0 (*p* < 0.001). On day 21, C14:0 was highest in the BSFL group, intermediate in the Mix group, and lower in the Control and FO groups (*p* < 0.05). Relative to baseline, C14:0 increased significantly in the BSFL and Mix groups, whereas no significant change was detected in the Control or FO groups.

A treatment × time interaction was also detected for iso-C16:0 (*p* = 0.013). Its proportion decreased significantly from baseline to day 21 in the FO, BSFL, and Mix groups, while no significant change was observed in the Control group. No between-treatment differences in iso-C16:0 were detected at either sampling time.

The proportion of C18:3 n-3 (ALA) decreased markedly over time (*p* < 0.001), with a significant treatment × time interaction (*p* = 0.001). ALA declined significantly from baseline to day 21 in all dietary groups, although no between-treatment differences were detected within either sampling time. Total n-3 polyunsaturated fatty acids (n-3 PUFA) showed a similar response, with a strong effect of time (*p* < 0.001) and a treatment × time interaction (*p* = 0.024); significant decreases were observed in all four groups.

Arachidonic acid (ARA) was affected by both treatment (*p* = 0.023) and time (*p* < 0.001), without a significant treatment × time interaction (*p* = 0.162). On day 21, ARA was higher in the Control group than in the FO group, whereas the BSFL and Mix groups were intermediate. Significant within-treatment decreases were detected in the FO and Mix groups.

Several additional fatty acid variables changed over time without evidence of a treatment-specific temporal response. C16:0 and C18:2 n-6 showed significant overall time effects (*p* = 0.034 and *p* = 0.006, respectively). C18:0 increased over time (*p* < 0.001), with significant within-treatment increases in the Control and FO groups. Eicosapentaenoic acid (EPA) and docosahexaenoic acid (DHA) decreased over time (both *p* < 0.001); EPA declined significantly in all four groups, whereas DHA declined significantly in the FO, BSFL, and Mix groups. Odd- and branched-chain fatty acids (OBCFA) and ΣLC-PUFA also decreased over time (both *p* < 0.001), with significant within-treatment decreases in FO, BSFL, and Mix. The n-6/n-3 ratio increased over time (*p* < 0.001), with significant increases in all four groups. No significant effects of treatment, time, or treatment × time were detected for saturated fatty acids (SFA), total PUFA, or C18:1 cis-9. The treatment effect for monounsaturated fatty acids (MUFA) did not meet the predefined significance threshold (*p* = 0.0502). Among the variables reported in Table 1, the treatment effect for C14:0 and the treatment × time interactions for C14:0 and ALA remained significant after FDR adjustment (Table S1).

### 2.2. Placental Tissue Fatty Acid Profile

The placental fatty acid profile was largely unaffected by maternal prepartum lipid supplementation (Table 2). Nevertheless, noticeable numerical differences were observed among dietary groups, particularly for PUFA and OBCFA, with generally lower proportions in the BSFL and Mix groups. No significant treatment effects were observed for the major individual fatty acids, including C14:0, C16:0, C16:1, C18:0, C18:1 cis-9, C18:2 n-6, arachidonic acid (ARA), or docosahexaenoic acid (DHA) (*p* > 0.05). Similarly, total SFA and MUFA did not differ among dietary treatments. A treatment effect was detected for iso-C15:0 (*p* = 0.017). Its proportion was lower in the BSFL group than in the Control group (*p* = 0.018), whereas the FO and Mix groups showed intermediate values and did not differ significantly from either group. Total PUFA differed significantly among treatments (*p* = 0.030), with lower mean values in the BSFL and Mix groups than in the Control and FO groups. However, none of the pairwise comparisons remained statistically significant after Holm adjustment. A similar pattern was observed for OBCFA (*p* = 0.047), with lower mean values in the BSFL and Mix groups, although no individual pairwise comparison reached statistical significance after correction for multiple comparisons. No significant treatment effects were observed for total n-6 PUFA or LC-PUFA (*p* = 0.158 and *p* = 0.138, respectively). Branched-chain fatty acids (BCFA) showed a numerical tendency toward lower values in the BSFL and Mix groups, but the overall treatment effect did not reach statistical significance (*p* = 0.086). None of the nominal treatment effects remained significant after FDR adjustment (Table S1).

### 2.3. Amniotic Fluid Fatty Acid Profile

The fatty acid composition of amniotic fluid showed limited treatment-related differences following maternal prepartum lipid supplementation (Table 3). Significant treatment effects were detected for C18:0, C20:0, and C20:1.

The proportion of C18:0 differed among dietary treatments (*p* = 0.032), with a higher value in the BSFL group than in the Control group (*p* = 0.035), whereas the FO and Mix groups showed intermediate values. C20:1 was also affected by treatment (*p* = 0.005). Its proportion was highest in the Mix group, which differed significantly from both the Control (*p* = 0.004) and FO groups (*p* = 0.023), while the BSFL group was intermediate.

A significant overall treatment effect was also observed for C20:0 (*p* = 0.037); however, none of the individual pairwise comparisons remained significant in the post hoc analysis.

No significant treatment effects were observed for the major fatty acid classes, including SFA, MUFA, PUFA, n-6 PUFA, n-3 PUFA, or LC-PUFA (*p* > 0.05). C17:1 showed a numerical difference among treatments, but the overall effect did not reach statistical significance (*p* = 0.054). None of the nominal treatment effects remained significant after FDR adjustment (Table S1).

### 2.4. Placental Histology and Bacterial Morphometry

Histological examination of placental tissue revealed preserved tissue architecture in all evaluated samples, irrespective of dietary treatment. The villous and cotyledonary epithelium was continuous and uniformly organized, with preserved cellular polarity. No epithelial desquamation, focal necrosis, edema, hypertrophy, or flattening of the epithelial layer was observed.

**Figure 1:**
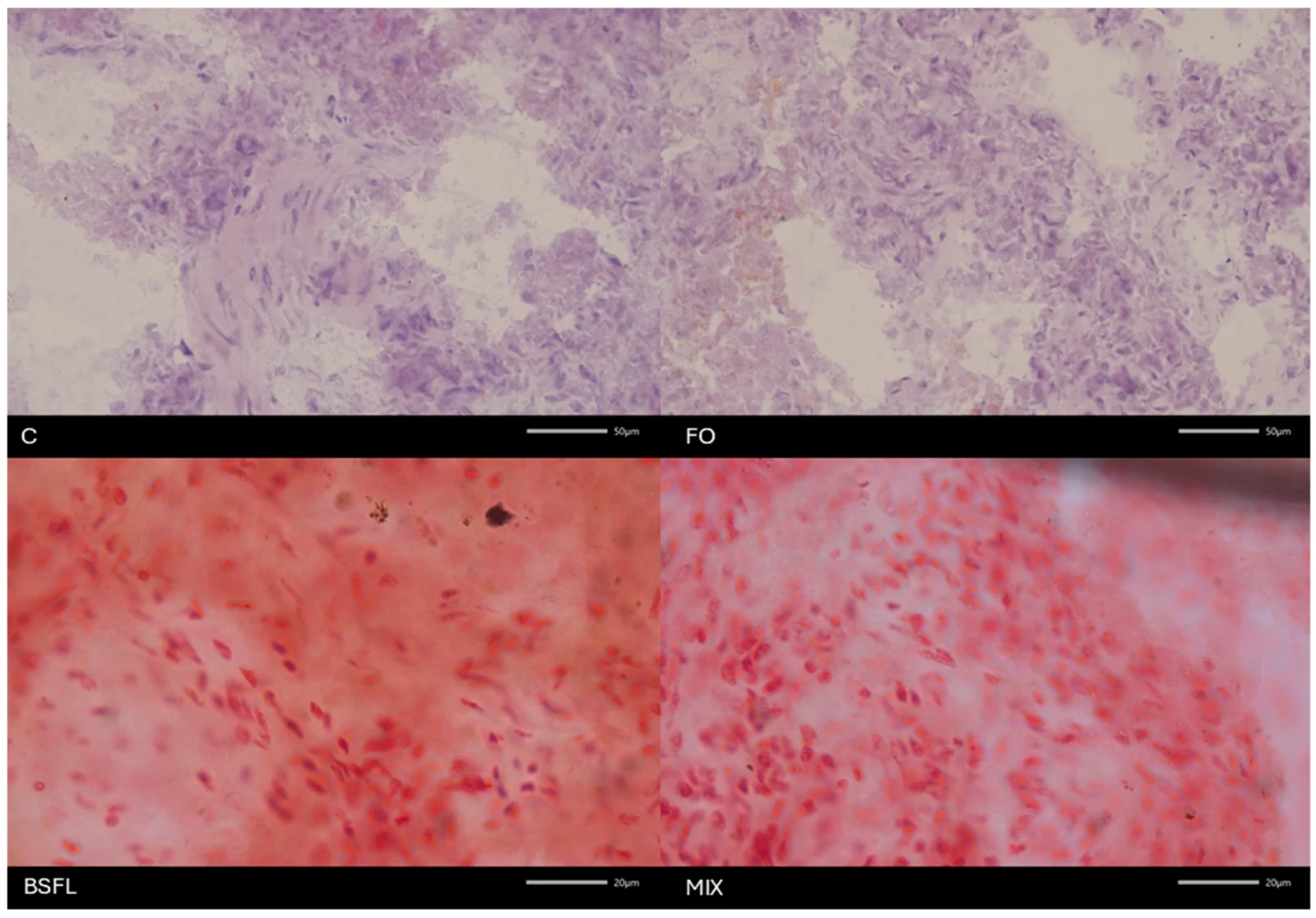
Representative histological appearance of ovine placental tissue following maternal dietary supplementation. H&E-stained paraffin-embedded placental sections showing preserved tissue architecture, intact villous and cotyledonary epithelium, normally organized stromal tissue, and no overt inflammatory, degenerative, or vascular lesions. Scale bars: Control and FO, 50 µm; BSFL and Mix, 20 µm. C, Control; FO, flaxseed oil; BSFL, black soldier fly larvae oil; Mix, flaxseed oil:BSFL oil blend (2:1).

The villous stroma showed regular organization, with normally distributed collagen fibers, fibroblasts, and fibrocytes. No evidence of fibrosis, scar-like remodeling, degenerative lesions, or reparative changes was identified. Placental blood vessels exhibited preserved endothelial integrity, with no apparent vascular dilation, congestion, or thrombus formation. No neutrophilic infiltration, focal lymphocytic accumulation, purulent lesions, or other histological signs of inflammation were detected. Overall, no treatment-associated pathological, inflammatory, degenerative, or vascular alterations were observed (Figure 1).

Gram-stained sections were additionally examined for the presence of structures exhibiting bacterial morphology within the deeper placental tissue. Only sparse bacterial-like structures were observed in these compartments, without evidence of bacterial microcolonies, purulent foci, tissue necrosis, inflammatory cell infiltration, or disruption of placental architecture. Thus, the presence of bacterial structures in deeper placental tissue was not accompanied by histopathological evidence of active infection. Bacterial structures associated with the placental surface were excluded from the analysis because their origin could not be reliably distinguished from environmental contamination occurring during or after spontaneous placental expulsion.

**Figure 2:**
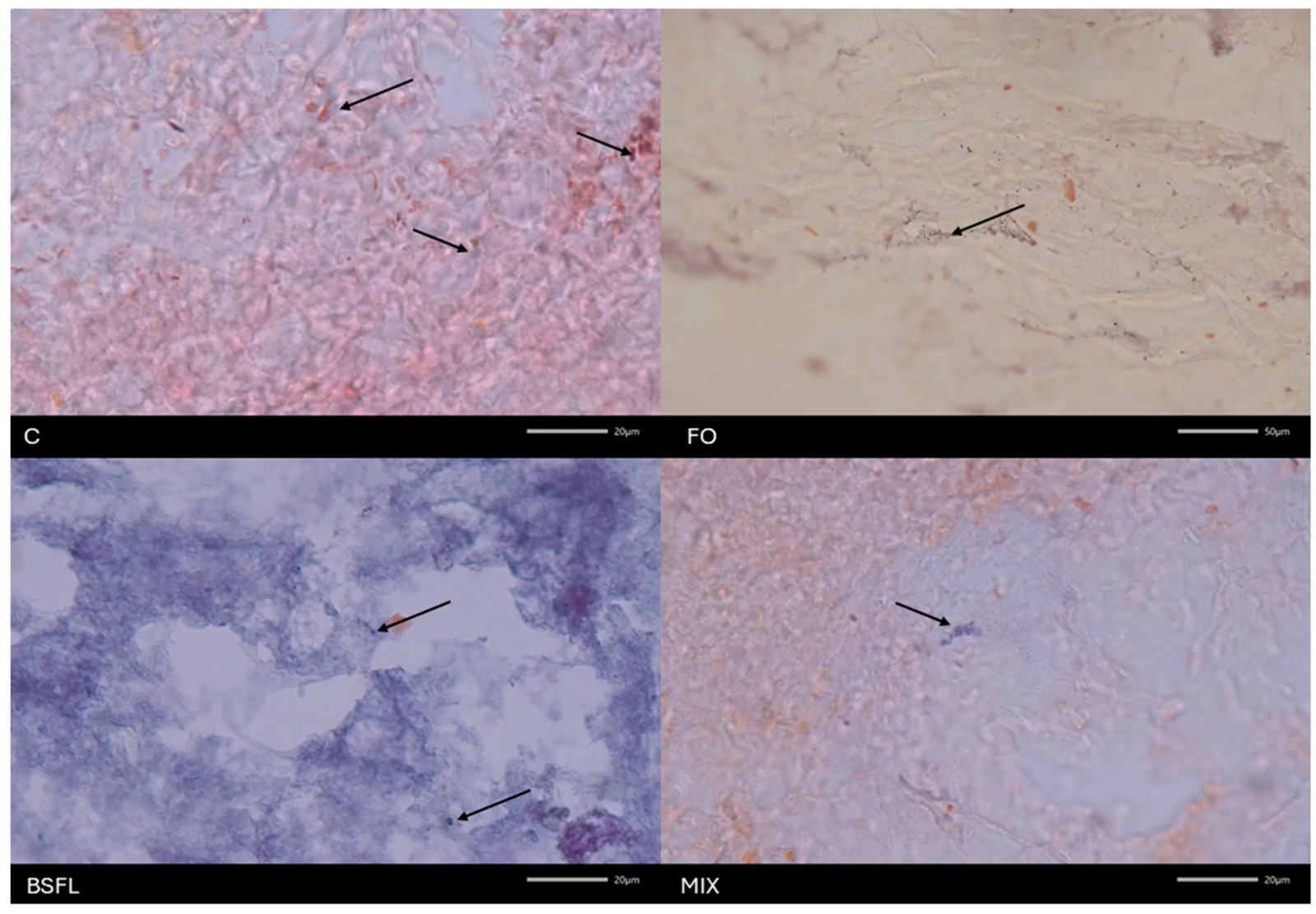
Bacterial-like structures observed in Gram-stained sections of deeper placental tissue. Gram stain, magnification 1000× (oil immersion). Representative Gram-stained sections of deeper placental tissue from the Control (C), FO, BSFL, and Mix groups. Arrows indicate sparse bacterial-like structures observed within the tissue. The bacterial structures were scattered and were not accompanied by inflammatory changes or tissue damage. C, Control; FO, flaxseed oil; BSFL, black soldier fly larvae oil; Mix, flaxseed oil:BSFL oil blend (2:1).

**Figure 3:**
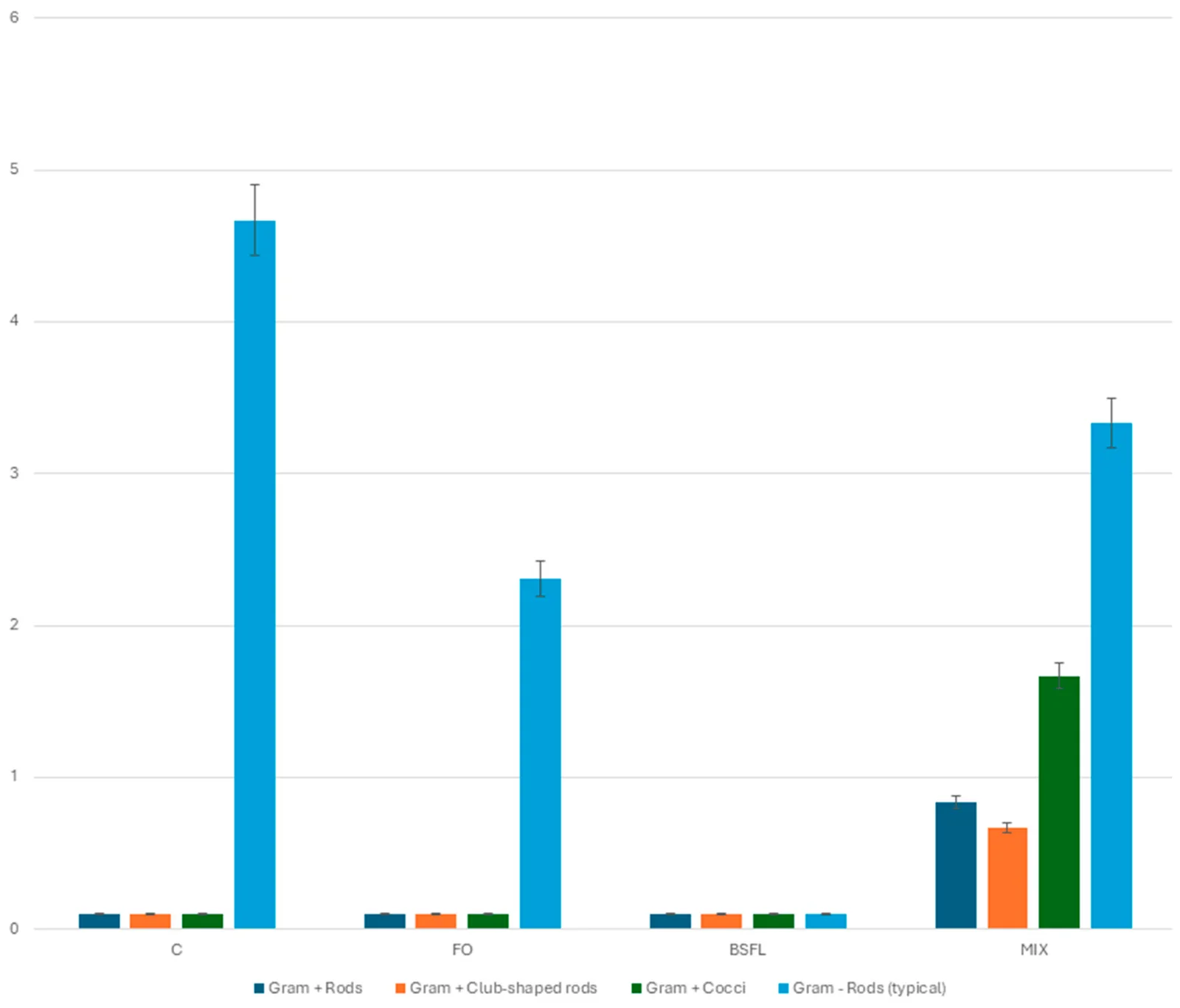
Morphometric profile of bacterial-like structures observed in Gram-stained sections of deeper placental tissue across the four dietary groups (C, FO, BSFL, Mix). Values are presented as mean ± SD of placenta-level mean counts, calculated from 10 consecutive microscopic fields per placenta for Gram-positive rods, Gram-positive club-shaped rods, Gram-positive cocci, and Gram-negative rods. The *y*-axis represents the mean number of bacterial structures per microscopic field. C, Control; FO, flaxseed oil; BSFL, black soldier fly larvae oil; Mix, flaxseed oil:BSFL oil blend (2:1).

Sparse bacterial-like structures were observed in Gram-stained sections of deeper placental tissue across all dietary groups (Figure 2). Morphometric analysis revealed no significant differences among dietary treatments (*p* > 0.05, Figure 3). The observed bacterial structures were not accompanied by inflammatory infiltration, tissue necrosis, or other histopathological evidence of active placental infection.

### 2.5. Lamb Serum Fatty Acid Profile

The serum fatty acid profile of lambs was strongly influenced by age, with additional treatment-related differences observed for selected fatty acids (Table 4). Nominal overall treatment effects (unadjusted *p* < 0.05) were detected for C12:0 (*p* = 0.017), C16:0 (*p* = 0.004), C18:0 (*p* = 0.005), C18:1 trans-11 (*p* = 0.005), ARA (*p* = 0.008), and DHA (*p* = 0.013).

At 2 days of age, C16:0 was higher in the FO and BSFL groups than in the Control group, whereas the Mix group showed an intermediate value. C18:1 trans-11 was higher in the Control group than in all three oil-supplemented groups. C14:0 was higher in the FO group than in the Control group, while DHA was higher in the Control group than in the Mix group, with FO and BSFL showing intermediate values.

At 21 days of age, C12:0 was higher in the BSFL group than in the Control, FO, and Mix groups. C18:0 was higher in the Control group than in the FO group, whereas the BSFL and Mix groups showed intermediate values. ARA was higher in the Mix group than in the BSFL group, with the Control and FO groups showing intermediate values.

Nominal treatment × age interactions were detected for total n-3 PUFA (*p* = 0.023) and the n-6/n-3 ratio (*p* = 0.020). For total n-3 PUFA, no pairwise treatment comparison within either age remained significant after Holm adjustment, although significant increases from day 2 to day 21 were observed in the FO and Mix groups. The n-6/n-3 ratio increased significantly between days 2 and 21 only in the BSFL group. The treatment × age interactions for DHA (*p* = 0.059) and LC n-3 PUFA (*p* = 0.052) did not meet the predefined significance threshold.

Age-related changes were observed for several individual fatty acids and fatty acid classes. C16:0 decreased significantly between days 2 and 21 in all four treatment groups. LA, ALA, ARA, total PUFA, total n-6 PUFA, and the PUFA/SFA ratio showed significant overall effects of age, with generally higher values at day 21. EPA was not significantly affected by treatment, age, or the treatment × age interaction (*p* > 0.05). After FDR adjustment, no treatment or treatment × age effects remained significant, whereas 27 of 45 age effects remained significant (Table S1).

### 2.6. Lamb Growth Performance

Maternal dietary treatment did not significantly affect lamb body weight at day 2 (*p* = 0.065), weaning weight at day 56 (*p* = 0.309), or overall average daily gain (*p* = 0.377) (Table 5). Numerically, lambs in the Mix group showed the highest adjusted mean weaning weight (15.90 kg) and ADG (223.26 g/day); however, these differences were not statistically significant. Survival to day 56 was 94.44% (17/18) in the Control group, 89.47% (17/19) in the FO group, 95.00% (19/20) in the BSFL group, and 89.47% (17/19) in the Mix group and was evaluated descriptively.

## 3. Discussion

Prepartum lipid supplementation was associated with compartment-specific changes in fatty acid composition across the maternal–placental–offspring axis. The maternal serum profile showed the clearest temporal and treatment-related variation, whereas placental tissue and amniotic fluid were comparatively less responsive. In maternal serum, several fatty acids and fatty acid groups changed markedly over the 21-day experimental period, including ALA, EPA, DHA, total n-3 PUFA, OBCFA, and the n-6/n-3 ratio. However, similar temporal changes were also observed in the Control group for several variables, indicating that part of the response was associated with the physiological and metabolic adaptations occurring during late gestation rather than with lipid supplementation alone. Previous studies in ewes have likewise demonstrated changes in circulating fatty acid profiles following lipid supplementation during late pregnancy [24,25,26]. Importantly, since the experimental oils differed in their overall fatty acid composition, the observed treatment-related responses cannot be attributed to individual fatty acids. Rather, they should be interpreted as effects associated with the respective lipid sources, without establishing the specific contribution of individual fatty acids or their underlying metabolic mechanisms.

Placental fatty acid composition remained comparatively stable despite changes observed in maternal serum. Most major individual fatty acids and the principal fatty acid classes were not significantly affected by maternal dietary treatment. Treatment-related differences were restricted to selected minor components, including iso-C15:0, while the overall effects detected for PUFA and OBCFA were not followed by significant pairwise differences after adjustment for multiple comparisons. Thus, placental fatty acid composition did not simply mirror that of the maternal circulation. This pattern is consistent with previous reports describing placental fatty acid uptake, intracellular metabolism, storage, and transfer to the fetus [20,21,22,23]. However, because placental fatty acid transport and lipid-metabolism pathways were not directly assessed in the present study, these mechanisms remain possible explanations rather than demonstrated findings. The absence of treatment-associated inflammatory, degenerative, or vascular lesions in the histological examination further indicates that none of the lipid supplements produced overt structural alterations in placental tissue.

A similarly selective response was observed in amniotic fluid. Treatment effects were identified for individual fatty acids, including C18:0, C20:0, and C20:1, whereas the overall proportions of SFA, MUFA, PUFA, n-6 PUFA, n-3 PUFA, and LC-PUFA remained unchanged. Thus, the treatment-related changes detected in maternal serum were not reproduced proportionally in the amniotic fluid fatty acid profile. This observation may be consistent with selective placental fatty acid handling [20,22,24]; however, fetal metabolism and fetal contributions to amniotic fluid composition may also play a role, and the underlying mechanisms cannot be determined from the present data. Moreover, because amniotic fluid represents a complex fetal compartment and is not equivalent to fetal plasma, differences in its fatty acid composition should not be interpreted as direct measurements of transplacental fatty acid transfer.

The serum fatty acid profile of the lambs provided an additional postnatal perspective. Age appeared to exert a particularly strong influence between days 2 and 21 of life, with increases in LA, ALA, ARA, total PUFA, n-3 PUFA, and n-6 PUFA and concurrent decreases in several saturated and monounsaturated fatty acids. Nevertheless, selected treatment-related differences were observed for C12:0, C16:0, C18:0, C18:1 trans-11, ARA, and DHA, while nominal treatment × age interactions were detected for total n-3 PUFA and the n-6/n-3 ratio, whereas the interactions for DHA and LC n-3 PUFA did not reach statistical significance. These findings indicate that postnatal age was strongly associated with changes in the circulating fatty acid profile, whereas treatment-related differences observed for selected fatty acids were nominal and did not remain significant after FDR adjustment. Previous studies have demonstrated that maternal fatty acid supplementation during late gestation can alter fatty acid profiles not only in maternal plasma but also in colostrum, milk, and lamb circulation [25,26]. Importantly, the first lamb blood samples in the present study were collected at 2 days of age, after colostrum intake. Consequently, the neonatal serum fatty acid profile cannot be attributed exclusively to prenatal placental transfer. Although maternal dietary treatments were applied only before parturition, treatment-associated differences in lamb serum cannot be assigned specifically to prenatal exposure, because maternal supplementation may also have influenced the fatty acid composition of colostrum and milk. It likely represents the combined effects of prenatal exposure, colostrum-derived fatty acids, and early postnatal lipid metabolism. By day 21, milk intake and age-related metabolic development are likely to contribute even more strongly to the circulating fatty acid profile. This interpretation is supported by previous studies examining the effects of maternal lipid supplementation on fatty acid composition in maternal and offspring compartments [24,25,26], as well as by our previous findings obtained using the same lipid sources [31]. These findings should also be considered in relation to our previous study using the same experimental model, in which prepartum supplementation with flaxseed oil, BSFL oil, and their blend altered the fatty acid composition of sheep milk [31]. While treatment effects on the major fatty acid classes were limited in transitional milk, more pronounced changes were observed in mature milk, including a reduction in total SFA, increased MUFA proportions in the BSFL and Mix groups, and higher proportions of cis-9, trans-11 conjugated linoleic acid (CLA) following FO and BSFL supplementation. Importantly, these differences remained detectable after supplementation had ceased at lambing, suggesting a carry-over effect of maternal prepartum nutrition on subsequent milk lipid composition. The lower proportion of C18:1 trans-11 (vaccenic acid) in the serum of 2-day-old lambs from supplemented ewes may be related to changes in maternal ruminal biohydrogenation and the subsequent availability of this fatty acid through colostrum [25,26,28,29]. Vaccenic acid is an intermediate of ruminal biohydrogenation and a precursor of cis-9, trans-11 CLA synthesis via Δ9-desaturase activity. In our previous study using the same experimental model, maternal lipid supplementation altered the fatty acid composition of sheep milk, including cis-9, trans-11 CLA [31]. However, circulating CLA proportions in lambs did not differ significantly among dietary treatments. Furthermore, the relative contributions of prenatal fatty acid transfer and postnatal colostrum intake could not be distinguished. Consequently, the observed differences in vaccenic acid should be interpreted as treatment-associated changes in fatty acid composition rather than direct evidence of altered biohydrogenation or endogenous CLA synthesis. These findings provide additional context for interpreting the treatment- and age-related differences observed in lamb serum. Changes in maternal milk fatty acid composition may have contributed to the offspring fatty acid profiles during early postnatal development, particularly at day 21. However, because pre-suckling blood samples were not collected, the relative contributions of prenatal fatty acid transfer, milk intake, and postnatal lipid metabolism cannot be distinguished.

Despite the observed changes in fatty acid composition, maternal lipid supplementation did not significantly affect lamb body weight or average daily gain. Survival to weaning varied numerically among dietary groups but was evaluated descriptively; therefore, no inferential conclusions regarding treatment effects on survival were drawn. Lambs in the Mix group showed numerically higher mean weaning weight and average daily gain, but these differences were not statistically significant and should therefore be interpreted cautiously. The lack of significant growth responses suggests that the biochemical changes in fatty acid composition did not translate into detectable differences in early postnatal growth under the conditions of the present experiment. Together with the absence of treatment-associated histological alterations in the placenta, these findings indicate that the functional significance of the observed differences in fatty acid composition remains uncertain.

Histological examination showed preserved placental architecture across all treatment groups, with no evidence of treatment-associated inflammatory infiltration, necrosis, edema, degenerative changes, or vascular lesions. Only sparse bacterial-like structures were observed in Gram-stained sections from deeper placental tissue, and these structures were not accompanied by inflammatory or necrotic lesions or other histopathological evidence of active infection. However, these observations do not establish bacterial identity, viability, stable colonization, or the presence of an intrinsic placental microbiota, and contamination during parturition, placental expulsion, or sample processing cannot be excluded.

Overall, the findings partially supported the original hypothesis, revealing distinct fatty acid patterns across the examined biological compartments. Maternal serum exhibited the most pronounced treatment-related changes in fatty acid composition, whereas placental tissue and amniotic fluid showed more limited responses, primarily involving selected fatty acids and fatty acid groups. In offspring serum, postnatal age was the predominant factor associated with fatty acid composition, whereas treatment-related differences detected for selected fatty acids were nominal and did not remain significant after FDR adjustment. Collectively, these findings indicate that changes in maternal fatty acid status are not uniformly reflected across the maternal–placental–offspring axis. The distinct fatty acid profiles observed in individual compartments may reflect differences in placental lipid handling, fetal metabolism, and postnatal nutrient supply, although the relative contributions of these processes cannot be determined from the present data. Several limitations should be considered when interpreting these findings. The relatively small number of ewes per treatment group and the limited availability of placental and amniotic fluid samples may have reduced the statistical power to detect treatment-related differences. After FDR adjustment, treatment-related findings in placental tissue, amniotic fluid, and lamb serum did not remain significant and should therefore be considered exploratory. The inclusion of only complete, quality-eligible placental tissue and amniotic fluid sample pairs may also have introduced selection bias, as non-random sample loss cannot be excluded. Furthermore, the absence of pre-suckling lamb blood samples precluded differentiation between prenatal fatty acid transfer and the early postnatal effects of colostrum intake. The lack of direct measurements of placental fatty acid transport, transporter expression, and lipid-metabolism pathways also limits mechanistic interpretation of the observed compartment-specific responses. Moreover, the present study involved comparisons of relative fatty acid composition in biological compartments sampled at different time points: maternal serum before parturition, placental tissue and amniotic fluid at parturition, and lamb serum on days 2 and 21 of life. Fetal blood was not collected, and no tracer-based measurements of fatty acid transfer were performed. Consequently, the present findings do not allow conclusions regarding the direction, magnitude, or causality of maternal-to-offspring fatty acid transfer. Future studies combining fatty acid profiling with placental transporter expression, umbilical cord blood sampling, stable isotope tracers, and pre-suckling neonatal blood sampling may help clarify the relative contributions of placental lipid handling and early postnatal nutrient supply to offspring fatty acid composition.

## 4. Materials and Methods

### 4.1. Ethical Procedure

All procedures involving animals were approved by the Local Ethical Committee for Animal Experimentation in Wrocław, Poland (Resolution No. 064/2023/P1 of 6 December 2023). The animals were maintained and handled in accordance with Directive 2010/63/EU on the protection of animals used for scientific purposes. No animals were euthanized specifically for the purposes of the present study. Placental tissue and fetal fluid samples were collected during spontaneous parturition, whereas blood samples were collected as part of the previously approved experimental protocol.

### 4.2. Animals and Experimental Design

The study was carried out at the Research and Teaching Station of Wrocław University of Environmental and Life Sciences in Swojczyce, Poland. No hormonal estrus synchronization was applied before breeding. Thirty-two clinically healthy pregnant Olkuska ewes, aged 3–5 years and with an average body weight of 65 ± 5 kg, were included in the experiment. Pregnancy status and fetal number were assessed by ultrasonography, and only ewes carrying two or three fetuses were eligible. Before randomization, animals were matched according to similar biological characteristics and subsequently randomly allocated to four dietary treatments (n = 8 per group). The control group received the basal diet without additional lipid supplementation, whereas the remaining groups received high-linoleic, low-ALA flaxseed oil (FO), black soldier fly larvae oil (BSFL; *Hermetia illucens*), or a 2:1 mixture of flaxseed and BSFL oils (Mix). The 2:1 blending ratio was selected to combine the contrasting fatty acid profiles of the two oils within a single experimental treatment, while maintaining a constant total supplementation level of 30 g/ewe/day. The BSFL oil was a commercially available insect-derived lipid product supplied by Grinsect, BSF Systems Kft., Hungary, used as a separated oil fraction rather than insect meal or processed animal protein (PAP).

The ewe was considered the experimental unit. Before the start of the supplementation period, all animals were assessed for general health and pregnancy status, and information on age, body weight, body condition score, litter size, lambing date, lamb sex, and birth weight was recorded. All ewes received the same basal diet, comprising meadow hay offered ad libitum and a custom concentrate containing 50% oats, 30% triticale, 10% soybean meal, 5% maize, and 5% mineral–vitamin premix. The concentrate allowance was 0.5 kg per ewe twice daily, irrespective of the number of fetuses, with unrestricted access to fresh water. The feeding regimen and detailed chemical and fatty acid composition of the basal diet have been described previously [31]. During the 21 days preceding the expected date of parturition, supplemented ewes received 30 g/day of the assigned lipid source: high-linoleic, low-ALA flaxseed oil in the FO group, BSFL oil in the BSFL group, or a 2:1 flaxseed-to-BSFL oil blend in the Mix group. The supplementation level was selected taking into account preliminary in vitro screening experiments evaluating the effects of different lipid sources and inclusion levels on ruminal fermentation and fatty acid profiles. These investigations informed the selection of the experimental dose without constituting a direct quantitative dose conversion. The same total oil supplementation level was maintained across treatments to ensure comparability. The oils were administered individually to ensure complete consumption, and supplementation was discontinued at lambing. The flaxseed oil (LinumVital^®^, Oleofarm, Wrocław, Poland) was characterized by a high proportion of linoleic acid and a low proportion of α-linolenic acid, whereas BSFL oil (Grinsect, BSF Systems Kft., Hódmezővásárhely, Hungary) was rich in saturated fatty acids, particularly lauric acid. The detailed fatty acid composition of the oils and basal diet components was determined by gas chromatography and has been reported previously. In the flaxseed oil used in this experiment, linoleic acid (C18:2 n-6) accounted for 54.66% of total identified fatty acids, whereas α-linolenic acid (C18:3 n-3) accounted for 2.96%. The BSFL oil was characterized by a high proportion of lauric acid (C12:0; 51.93%) [31]. Maternal serum fatty acid analyses included all 32 ewes (n = 8 per treatment group), whereas placental tissue and amniotic fluid fatty acid analyses were performed on 24 samples of each biological material (n = 6 per treatment group). The exact sample numbers used in each analysis are reported in the corresponding tables.

### 4.3. Sample Protocol

#### 4.3.1. Maternal and Lamb Blood Sampling

Maternal blood samples were collected from the jugular vein at two time points: immediately before the start of dietary supplementation, which was considered the baseline measurement, and after 21 days of prepartum supplementation, before parturition. Blood samples were collected in the morning before the first feeding. Maternal serum fatty acid profiles were determined for eight ewes per dietary treatment (n = 8 per group). Blood samples from lambs were collected at 2 and 21 days of age by jugular venipuncture. The first lamb sampling therefore took place after colostrum intake; pre-suckling and umbilical cord blood samples were not collected. At each sampling, approximately 4 mL of blood was collected into sterile serum tubes containing a clot activator (SARSTEDT, Warsaw, Poland). Within 2 h of collection, the samples were centrifuged at 3000× *g* for 10 min at room temperature. The separated serum was transferred into labeled microcentrifuge tubes and stored at −20 °C until fatty acid analysis. The broader experimental programme also included maternal biochemical and hematological analyses and the determination of total antioxidant status (TAS). The biochemical and antioxidant findings obtained from the same experimental animals were reported previously [27].

#### 4.3.2. Placental Tissue Collection

Placentas were collected as soon as possible after spontaneous expulsion following delivery of the last lamb. Placental tissue was sampled separately according to the intended subsequent analysis. For fatty acid determination, three macroscopically intact fetal cotyledons were collected from each placenta, representing the peripheral region, the central region, and the area close to the umbilical cord insertion. Cotyledons showing visible maceration, marked mechanical damage, abnormal discoloration, or adherent blood clots were excluded. The three cotyledons were pooled to obtain one composite sample per ewe, placed in a sterile container, and immediately frozen at −20 °C until further processing. Fatty acid composition was determined in six composite placental samples per dietary treatment (n = 6 per group; 24 samples in total).

Separate placental tissue fragments were collected for histological and bacteriological examination. Tissue intended for histological evaluation was fixed immediately after collection and processed separately for routine paraffin histology. Placental tissue intended for bacterial morphometry was frozen and subsequently processed using cryostat sectioning. Placental weight and surface area were not determined, as the placental assessment focused on fatty acid composition and histological characteristics rather than quantitative measures of placental transport efficiency.

#### 4.3.3. Amniotic Fluid Collection

Amniotic fluid was collected during parturition by sterile puncture of the first intact amniotic sac presenting in the birth canal and visibly containing the first lamb. The sample was obtained before spontaneous rupture of the fetal membranes and transferred into sterile polypropylene tubes. Each sample was visually inspected after collection. Samples showing visible blood contamination, fecal-like particulate material, or external debris (e.g., bedding) were excluded from further analysis. The remaining samples were frozen at −20 °C and stored until lipid extraction. Fatty acid composition was determined in six amniotic fluid samples per dietary treatment (n = 6 per group; 24 samples in total). Only complete pairs of placental tissue and amniotic fluid samples originating from the same ewe and meeting the required quality and quantity criteria were included in the fatty acid analyses, resulting in six matched sample pairs per dietary treatment.

### 4.4. Laboratory Analyses

#### 4.4.1. Lipid Extraction from Maternal and Lamb Serum Samples

Serum samples were thawed at room temperature immediately before analysis. For fatty acid determination, 150 µL of each serum sample was transferred into an individual microcentrifuge tube. Prior to lipid extraction, each sample was spiked with 10 µg of undecanoic acid (C11:0), added as 50 µL of a 0.2 mg/mL solution in methanol and used as an internal standard.

Total lipids were extracted using 500 µL of chloroform and 250 µL of methanol, corresponding to a chloroform:methanol ratio of 2:1 (*v*/*v*), according to the Folch method. The samples were vortexed for 30 s and subsequently centrifuged at 14,500× *g* for 4 min. Following phase separation, the lower chloroform phase containing the lipid fraction was carefully collected and evaporated under gentle heating. The obtained lipid fraction was subsequently subjected to saponification and methylation.

#### 4.4.2. Preparation and Lipid Extraction from Placental Tissue

Placental tissue samples were frozen immediately after collection and subsequently lyophilized for approximately 5 days until completely dry. The lyophilized tissue was homogenized to obtain a uniform fine powder. For fatty acid analysis, 0.500 g of each homogenized sample was accurately weighed and transferred into an individual extraction vessel. Prior to lipid extraction, each sample was spiked with 10 µg of heptadecanoic acid (C17:0), added as 50 µL of a 0.2 mg/mL solution in methanol and used as an internal standard.

Placental lipids were extracted with 13 mL of chloroform:methanol (2:1, *v*/*v*) according to the Folch method. After 30 s of vortex mixing and centrifugation at 14,500× *g* for 4 min, the chloroform–methanol extract containing the lipid fraction was carefully recovered, leaving residual tissue material in the extraction vessel, and evaporated under gentle heating. The obtained lipid fraction was subsequently subjected to saponification and methylation.

#### 4.4.3. Preparation and Lipid Extraction from Amniotic Fluid

For fatty acid determination, amniotic fluid was collected at parturition into sterile 150 mL containers. Immediately after collection, the samples were divided into 50 mL aliquots and transferred into sterile 100 mL tubes. The samples were subsequently frozen and lyophilized for 5 days until completely dry. The lyophilized material was homogenized to obtain a uniform fine powder. Subsequently, 100 mg of each homogenized sample was accurately weighed into individual extraction vessels. Prior to lipid extraction, each sample was spiked with 10 µg of heptadecanoic acid (C17:0), added as 50 µL of a 0.2 mg/mL solution in methanol and used as an internal standard.

Amniotic-fluid lipids were extracted with 2 mL of chloroform:methanol (2:1, *v*/*v*) according to the Folch method. Vortexing, centrifugation, recovery of the chloroform–methanol extract containing the lipid fraction while leaving residual non-lipid material in the extraction vessel, and evaporation under gentle heating were performed as described above for placental samples.

#### 4.4.4. Preparation of Fatty Acid Methyl Esters and Chromatographic Analysis

The extracted lipid fractions were subjected to saponification and methylation for the preparation of fatty acid methyl esters (FAME). For placental tissue and amniotic fluid samples, 2 mL of 1 M potassium hydroxide solution in methanol was added to the lipid extract. For serum samples, 300 µL of 1 M potassium hydroxide solution in methanol was used. The mixtures were heated at 80 °C for 3 min.

Subsequently, 1.5 mL of 14% boron trifluoride solution (BF_3_) was added to placental tissue and amniotic fluid samples, whereas 100 µL was added to serum samples. The mixtures were heated again at 80 °C for 3 min.

After heating, 0.5 mL of distilled water and 0.5 mL of saturated aqueous NaCl solution were added to placental tissue and amniotic fluid samples. In serum samples, 50 µL of saturated aqueous NaCl solution was added without the addition of distilled water. Subsequently, 5 mL of cyclohexane was added to placental tissue and amniotic fluid samples, whereas 300 µL was added to serum samples.

The samples were vortexed for 30 s and centrifuged at 14,500× *g* for 4 min. The upper cyclohexane phase containing FAME was carefully collected and filtered through a layer of cotton and Celite. The filtrate was transferred into an evaporation vessel and evaporated to dryness. The residue was subsequently dissolved in 300 µL of cyclohexane and transferred into a 300 µL glass insert for chromatographic analysis.

FAMEs were analyzed on a Shimadzu GCMS-QP2020 gas chromatography–mass spectrometry system (Shimadzu, Kyoto, Japan) equipped with a Zebron ZB-FAME capillary column (60 m × 0.25 mm × 0.20 µm; Phenomenex, Torrance, CA, USA). The injector was maintained at 280 °C with a split ratio of 1:80. The oven program started at 80 °C (2 min), followed by ramps of 3 °C min^−1^ to 180 °C and 8 °C min^−1^ to 240 °C, with a final 4 min hold. Helium was used as the carrier gas at 1.80 mL min^−1^.

Mass spectra were acquired in scan mode over m/z 40–400 at 3 scans s^−1^, with ion-source and interface temperatures of 220 °C and 250 °C, respectively. Fatty acids were identified by spectral comparison with the NIST 20 library and by retention-time matching against the Supelco 37 Component FAME Mix (Sigma-Aldrich, St. Louis, MO, USA). Individual fatty acids were expressed as percentages of total identified fatty acids, from which fatty acid classes and ratios were calculated. Internal standards (C11:0 for serum and C17:0 for placental tissue and amniotic fluid) were excluded before the remaining endogenous fatty acids were renormalized to 100%.

#### 4.4.5. Histological Examination of Placental Tissue

Placental tissue samples intended for histological examination were fixed immediately after collection and processed using routine histological procedures. Following fixation, representative tissue fragments were dehydrated through a graded series of ethanol, cleared, embedded in paraffin, and sectioned at 7 µm thickness using a microtome. The sections were mounted on glass slides and stained with hematoxylin and eosin (H&E). Histological sections were examined using a Nikon Eclipse 80i light microscope (Nikon, Japan).

Placental morphology was evaluated in representative microscopic fields from each tissue sample. The assessment included general tissue architecture and the presence of histopathological alterations, including vascular changes, inflammatory cell infiltration, hemorrhage, edema, tissue degeneration, and necrotic lesions. Observations obtained from multiple microscopic fields within the same placenta were treated as technical observations of the same biological sample and were combined to obtain a representative assessment for each ewe.

#### 4.4.6. Morphometric Assessment of Bacterial Morphotypes in Placental Tissue

Frozen placental tissue samples were sectioned using a cryostat maintained at −20 °C to obtain 10 µm-thick sections. Tissue sections were stained using a modified Gram staining procedure and examined using a Nikon Eclipse 80i light microscope (Nikon, Japan). Bacterial structures were classified according to their Gram-staining properties and morphology as Gram-positive rods, Gram-positive club-shaped rods, Gram-positive cocci, and Gram-negative rods.

Bacterial morphotypes were assessed in deeper placental tissue. For each tissue section, bacterial structures were counted in 10 consecutive microscopic fields examined at 1000× magnification using oil immersion. Counts obtained from multiple microscopic fields were considered technical measurements of the same biological sample. This assessment was descriptive, as Gram staining and morphological classification do not establish bacterial identity, viability, or stable colonization of placental tissue. Culture-based or molecular identification was not performed.

#### 4.4.7. Lamb Growth Performance

Lamb rearing performance was evaluated from the second day of life until weaning. All live-born lambs included in the postnatal follow-up were individually identified and reared by their dams under standard flock management conditions. No artificial rearing, milk replacer supplementation, or additional milk feeding was applied during the rearing period.

Lamb body weight was recorded on day 2 postpartum, with the day of birth considered as day 1, and again at weaning on day 56. Average daily gain (ADG) was calculated individually for each lamb over the 54-day period between day 2 and day 56 according to the following formula:

ADG = (body weight at day 56 − body weight at day 2)/54(1)

Lamb survival was monitored from birth until weaning and reported descriptively as the number of lambs alive at Day 56 relative to the number of live-born lambs included in postnatal follow-up in each treatment group [n/N (%)]. Stillborn lambs were excluded from the postnatal growth and survival analyses. Mortality was recorded during the rearing period; however, causes of lamb death were not systematically classified and therefore were not included in the statistical analysis. Lamb sex and litter size were included in the statistical model to account for their potential effects on growth performance.

### 4.5. Statistical Analysis

Statistical analyses were performed using Statistica software, version 13.0 (TIBCO Software Inc., Palo Alto, CA, USA), and Python version 3.13.5 with the statsmodels package (version 0.14.6). Linear mixed-effects models for lamb serum fatty acid profiles and lamb growth outcomes were fitted using the MixedLM implementation in statsmodels. Statistical analyses were performed separately for maternal serum, placental tissue, amniotic fluid, lamb serum, and lamb growth outcomes. The ewe was considered the experimental unit for maternal and placental analyses. Statistical significance was accepted at *p* < 0.05.

Maternal serum fatty acid data were analyzed using a repeated-measures model including dietary treatment, sampling time, and the treatment × time interaction as fixed effects, with ewe included as the repeated subject nested within treatment. Because two sampling times were evaluated, no sphericity correction was required. When appropriate, differences among dietary treatments within the same sampling time were examined using Tukey’s HSD test. Changes between baseline and day 21 within individual treatment groups were evaluated using paired comparisons with Holm adjustment for multiple testing. Results are presented as treatment × time means with pooled SEM.

Placental and amniotic-fluid fatty acid data were analyzed independently for each fatty acid and derived fatty acid group. Residual normality was assessed using the Shapiro–Wilk test and homogeneity of variance using the Brown–Forsythe test. Variables meeting the assumptions of normality and homoscedasticity were analyzed by one-way ANOVA followed by Tukey’s HSD test. Welch’s ANOVA followed by the Games–Howell test was used when variance homogeneity was not satisfied. When residuals were non-normally distributed, natural-log transformation was evaluated; variables that remained non-normal were analyzed using the Kruskal–Wallis test followed, when significant, by Dunn’s pairwise test with Holm adjustment. Arithmetic means and pooled SEM are presented for consistency across tables, whereas *p*-values correspond to the statistical test selected for each variable.

Lamb serum fatty acid data were analyzed using a linear mixed-effects model including maternal dietary treatment, age (day 2 and day 21), and the treatment × age interaction as fixed effects. Dam (ewe) was included as a random intercept, with lamb identity nested within the dam as an additional random intercept to account for clustering of siblings and repeated measurements within the same lamb. The mixed-effects approach allowed all available observations to be retained, including lambs for which only one of the two sampling points was available. Pairwise treatment contrasts within each age and within-treatment age contrasts were adjusted for multiple comparisons using the Holm method. Results are presented as arithmetic treatment × age means with pooled model-based standard errors. Statistical significance was accepted at *p* < 0.05.

Lamb body weight at day 2, weaning weight at day 56, and average daily gain were analyzed using linear mixed-effects models including maternal dietary treatment, lamb sex, and litter size as fixed effects, with dam included as a random intercept to account for clustering of littermates. Results are presented as least-squares means (LSM) ± model-based standard error (SE). Pairwise treatment contrasts were adjusted for multiple comparisons using the Holm method. Lamb survival to weaning was summarized descriptively as n/N (%), without inferential statistical comparison among dietary treatment groups.

To account for multiple fatty acid endpoints, the Benjamini–Hochberg false discovery rate (FDR) procedure was applied to omnibus *p*-values separately for each model term within each biological compartment. The adjustment included all tested individual fatty acids and derived indices. FDR-adjusted *p*-values (q-values) are provided in Supplementary Table S1. Findings with *p* < 0.05 but q ≥ 0.05 were considered nominal and interpreted as exploratory.

For placental bacterial morphometry, counts obtained from multiple microscopic fields within the same placenta were treated as technical measurements and averaged to provide one value per bacterial morphotype and placenta. The ewe/placenta, rather than the individual microscopic field, constituted the experimental unit. Histopathological findings without quantitative scoring were reported descriptively.

## 5. Conclusions

Responses to prepartum lipid supplementation differed among biological compartments, with maternal serum showing greater variation than placental tissue or amniotic fluid. Lamb serum fatty acid composition was strongly influenced by postnatal age, whereas nominal treatment-associated differences did not remain significant after FDR adjustment. Placental histology revealed no treatment-associated inflammatory, degenerative, or vascular lesions. Maternal lipid supplementation did not significantly affect lamb growth performance. Taken together, changes in maternal fatty acid status were not consistently paralleled by corresponding changes in placental, amniotic-fluid, and offspring fatty acid profiles. Because placental fatty acid transport was not directly assessed and lamb serum was sampled after colostrum intake, these findings describe compartment-specific differences in relative fatty acid composition rather than demonstrating transplacental transfer. The mechanisms underlying these responses, including the possible roles of placental lipid handling and postnatal nutrition, require further investigation.

## Figures and Tables

**Table 1:** Effects of prepartum lipid supplementation on the maternal serum fatty acid profile before and after 21 days of supplementation (% of total identified fatty acids).

Fatty Acid/Group	Control		FO		BSFL		Mix		Pooled SEM	*p*-Value		
	Baseline	Day 21	Baseline	Day 21	Baseline	Day 21	Baseline	Day 21		Treatment	Time	Treatment × Time
C14:0	1.09	1.07 ^c^	1.18	1.01 ^c^	1.22	1.89 ^a†^	1.18	1.43 ^b†^	0.059	<0.001	<0.001	<0.001
C16:0	42.46	41.02	41.68	40.02	43.14	41.97	40.99	39.67	0.911	0.109	0.034	0.994
iso-C16:0	0.21	0.20	0.29	0.20 ^†^	0.29	0.20 ^†^	0.28	0.19 ^†^	0.023	0.403	<0.001	0.013
C18:0	34.61	36.28 ^†^	34.02	37.02 ^†^	33.22	35.17	35.79	37.46	0.923	0.210	<0.001	0.740
C18:1 cis-9	5.10	4.83	5.29	5.49	5.30	5.56	5.65	5.78	0.307	0.254	0.640	0.670
C18:2 n-6	8.31	9.07	7.63	8.71	7.90	8.15	7.48	8.38	0.482	0.573	0.006	0.693
C18:3 n-3 (ALA)	1.35	0.94 ^†^	1.80	1.08 ^†^	1.74	1.10 ^†^	1.51	1.20 ^†^	0.115	0.232	<0.001	0.001
C20:4 n-6 (ARA)	0.97	0.91 ^a^	0.78	0.65 ^b†^	0.90	0.72 ^ab^	0.97	0.71 ^ab†^	0.058	0.023	<0.001	0.162
C20:5 n-3 (EPA)	0.51	0.35 ^†^	0.52	0.37 ^†^	0.53	0.41 ^†^	0.55	0.40 ^†^	0.038	0.714	<0.001	0.849
C22:6 n-3 (DHA)	0.77	0.63	0.74	0.53 ^†^	0.79	0.52 ^†^	0.83	0.60 ^†^	0.053	0.674	<0.001	0.253
SFA	81.99	81.96	81.95	81.51	81.58	82.00	81.60	81.43	0.778	0.955	0.919	0.949
MUFA	5.85	5.86	6.77	6.99	6.72	7.22	6.81	7.09	0.418	0.050	0.365	0.937
PUFA	12.24	12.22	11.76	11.69	12.16	11.32	11.68	11.72	0.555	0.826	0.461	0.709
OBCFA	3.50	3.21	4.66	3.12 ^†^	3.68	2.64 ^†^	3.34	2.56 ^†^	0.435	0.382	<0.001	0.152
n-6 PUFA	9.50	10.22	8.61	9.54	9.01	9.13	8.67	9.25	0.520	0.469	0.037	0.750
n-3 PUFA	2.62	1.92 ^†^	3.07	1.98 ^†^	3.06	2.03 ^†^	2.89	2.20 ^†^	0.171	0.559	<0.001	0.024
ΣLC-PUFA	2.32	1.96	2.14	1.62 ^†^	2.31	1.75^†^	2.45	1.76 ^†^	0.135	0.406	<0.001	0.384
n-6/n-3	3.72	5.60 ^†^	2.92	5.12 ^†^	3.00	4.88 ^†^	3.04	4.25 ^†^	0.449	0.332	<0.001	0.463

Values are treatment × time means (equivalent to estimated marginal means in this complete balanced design) with pooled SEM; n = 8 ewes per treatment. Data were analyzed using a repeated-measures model including dietary treatment, time, and treatment × time as fixed effects, with ewe as the repeated subject nested within treatment. Within the same sampling time, means with different lowercase superscript letters differ significantly among dietary treatments (Tukey’s HSD test, *p* < 0.05). ^†^ indicates a significant difference from baseline within the same dietary treatment (paired post hoc comparison with Holm adjustment, *p* < 0.05). FO, flaxseed oil; BSFL, black soldier fly larvae oil; Mix, flaxseed oil:BSFL oil blend (2:1); ALA, α-linolenic acid; ARA, arachidonic acid; EPA, eicosapentaenoic acid; DHA, docosahexaenoic acid; SFA, saturated fatty acids; MUFA, monounsaturated fatty acids; PUFA, polyunsaturated fatty acids; OBCFA, odd- and branched-chain fatty acids; LC-PUFA, long-chain polyunsaturated fatty acids. n-6 PUFA = C18:2 cis n-6 + C18:3 n-6 + C20:3 n-6 + C20:4 n-6; n-3 PUFA = C18:3 n-3 + C20:5 n-3 + C22:6 n-3; ΣLC-PUFA = C20:3 n-6 + C20:4 n-6 + C20:5 n-3 + C22:6 n-3; OBCFA = anteiso-C15:0 + C15:0 + iso-C16:0 + anteiso-C16:0 + anteiso-C17:0 + C17:0 + iso-C18:0. BSFL oil was derived from *Hermetia illucens*.

**Table 2:** Effects of maternal prepartum lipid supplementation on the fatty acid profile of placental tissue (% of total identified fatty acids).

Fatty Acid/Group	Control (n = 6)	FO (n = 6)	BSFL (n = 6)	Mix (n = 6)	Pooled SEM	*p*-Value
C14:0	2.72	2.69	2.86	2.82	0.191	0.649
iso-C15:0	0.28 ^a^	0.22 ^ab^	0.09 ^b^	0.14 ^ab^	0.054	0.017
C16:0	33.30	32.94	34.21	33.83	1.718	0.955
C16:1 cis-9	1.24	1.33	1.17	1.20	0.111	0.436
C18:0	26.87	24.69	24.90	24.86	1.513	0.932
C18:1 cis-9	14.13	18.15	18.44	18.93	3.189	0.533
C18:2 n-6	2.44	2.19	1.68	1.97	0.314	0.395
C20:4 n-6 (ARA)	2.19	2.15	1.65	1.70	0.243	0.269
C22:6 n-3 (DHA)	1.50	1.40	1.18	0.95	0.201	0.213
SFA	70.74	67.75	68.79	68.50	3.257	0.990
MUFA	21.78	25.33	25.76	25.64	3.152	0.559
PUFA	7.48	6.92	5.45	5.86	0.639	0.030
BCFA	3.46	3.27	2.58	2.80	0.324	0.086
OBCFA	5.14	5.06	4.29	4.46	0.337	0.047
n-6 PUFA	5.52	5.23	3.93	4.55	0.504	0.158
n-3 PUFA	1.50	1.40	1.18	0.95	0.201	0.213
ΣLC-PUFA	4.51	4.13	3.37	3.22	0.444	0.138
n-6/n-3	4.20	3.89	3.58	5.70	0.699	0.162

Values are presented as arithmetic means with pooled SEM. C17:0, used as an internal standard, was excluded from the total fatty acid sum before calculation of relative fatty acid proportions, and the remaining identified fatty acids were renormalized to 100%. Distributional assumptions were assessed using the Shapiro–Wilk test of residuals and the Brown–Forsythe test for homogeneity of variance. Depending on data distribution, treatment effects were evaluated using one-way ANOVA, Welch’s ANOVA, or the Kruskal–Wallis test; logarithmic transformation was applied when appropriate. Tukey’s HSD, Games–Howell, or Dunn’s test with Holm adjustment was used for post hoc comparisons following a significant overall treatment effect. Within a row, means with different lowercase superscript letters differ significantly (*p* < 0.05). FO, flaxseed oil; BSFL, black soldier fly larvae oil (*Hermetia illucens*); Mix, flaxseed oil:BSFL oil blend (2:1); ARA, arachidonic acid; DHA, docosahexaenoic acid; SFA, saturated fatty acids; MUFA, monounsaturated fatty acids; PUFA, polyunsaturated fatty acids; BCFA, branched-chain fatty acids; OBCFA, odd- and branched-chain fatty acids; LC-PUFA, long-chain polyunsaturated fatty acids.

**Table 3:** Effects of maternal prepartum lipid supplementation on the fatty acid profile of amniotic fluid (% of total identified fatty acids).

Fatty Acid/Group	Control (n = 6)	FO (n = 6)	BSFL (n = 6)	Mix (n = 6)	Pooled SEM	*p*-Value
C12:0	0.94	0.97	1.26	1.12	0.303	0.857
C14:0	2.01	1.77	2.83	2.64	0.799	0.728
C16:0	17.28	30.80	31.81	35.07	4.996	0.132
C16:1	11.87	7.10	10.04	10.96	2.961	0.666
C17:1	26.24	12.29	10.44	5.37	4.770	0.054
C18:0	11.60 ^ab^	17.64 ^ab^	25.14 ^a^	23.35 ^ab^	3.128	0.032
C18:1 trans-9	3.40	6.62	7.58	8.25	2.165	0.210
C18:2 n-6	4.87	5.21	3.07	3.10	0.988	0.262
C20:0	6.64	5.18	1.51	1.12	1.445	0.037
C20:1	0.40 ^b^	0.60 ^b^	0.87 ^b^	2.14 ^a^	0.323	0.005
C21:0	9.67	7.58	3.09	3.86	1.988	0.103
C20:3 n-6	0.42	0.21	0.31	0.25	0.126	0.417
C22:0	4.38	3.46	1.51	2.18	1.055	0.337
SFA	52.52	67.40	67.16	69.34	4.654	0.167
MUFA	41.91	26.61	28.93	26.72	4.249	0.081
PUFA	5.57	5.99	3.91	3.94	0.994	0.301
n-6 PUFA	5.30	5.43	3.38	3.35	1.009	0.279
n-3 PUFA	0.27	0.57	0.53	0.59	0.228	0.550
ΣLC-PUFA	0.70	0.78	0.84	0.84	0.228	0.960

Values are presented as arithmetic means with pooled SEM. C17:0, used as an internal standard, was excluded from the total fatty acid sum before calculation of relative fatty acid proportions, and the remaining identified endogenous fatty acids were renormalized to 100%. Distributional assumptions were assessed using the Shapiro–Wilk test of residuals and the Brown–Forsythe test for homogeneity of variance. Depending on data distribution, treatment effects were evaluated using one-way ANOVA, Welch’s ANOVA, or the Kruskal–Wallis test; logarithmic transformation was applied when appropriate. Tukey’s HSD, Games–Howell, or Dunn’s test with Holm adjustment was used for post hoc comparisons following a significant overall treatment effect. Within a row, means with different lowercase superscript letters differ significantly (*p* < 0.05). C20:3 n-3 was the only explicitly identified n-3 PUFA in the amniotic-fluid dataset; therefore, n-3 PUFA corresponds to C20:3 n-3. FO, flaxseed oil; BSFL, black soldier fly larvae oil (*Hermetia illucens);* Mix, flaxseed oil:BSFL oil blend (2:1); SFA, saturated fatty acids; MUFA, monounsaturated fatty acids; PUFA, polyunsaturated fatty acids; LC-PUFA, long-chain polyunsaturated fatty acids.

**Table 4:** Effects of maternal prepartum lipid supplementation on the serum fatty acid profile of lambs at 2 and 21 days of age (% of total identified fatty acids).

Fatty Acid/Group	Control		FO		BSFL		Mix		Pooled SEM	*p*-Value		
	Day 2	Day 21	Day 2	Day 21	Day 2	Day 21	Day 2	Day 21		Treatment	Age	Treatment × Age
C12:0	1.25 ^a^	1.16 ^b^	1.57 ^a^	1.28 ^b^	1.32 ^a^	2.00 ^a^	1.06 ^a^	1.15 ^b^	0.190	0.017	0.461	0.053
C14:0	4.90 ^b^	4.58 ^a^	6.94 ^a^	4.74 ^a^	6.22 ^ab^	5.22 ^a^	5.32 ^ab^	4.34 ^a^	0.533	0.079	0.002	0.333
C16:0	36.92 ^b^	33.58 ^a^	41.22 ^a^	37.22 ^a^	41.51 ^a^	35.63 ^a^	39.53 ^ab^	34.78 ^a^	1.12	0.004	<0.001	0.721
C18:0	24.98 ^a^	27.99 ^a^	22.16 ^a^	24.14 ^b^	21.82 ^a^	24.52 ^ab^	24.17 ^a^	26.55 ^ab^	1.02	0.005	<0.001	0.946
C18:1 trans-11	1.15 ^a^	0.682 ^a^	0.539 ^b^	0.433 ^a^	0.515 ^b^	0.461 ^a^	0.553 ^b^	0.470 ^a^	0.122	0.005	0.023	0.224
C18:1 cis-9	13.43 ^a^	12.09 ^a^	12.23 ^a^	12.47 ^a^	13.67 ^a^	12.13 ^a^	13.35 ^a^	11.95 ^a^	0.539	0.858	<0.001	0.100
C18:2 cis-9,cis-12 (LA)	7.48 ^a^	9.63 ^a^	5.87 ^a^	8.81 ^a^	5.45 ^a^	9.20 ^a^	6.10 ^a^	10.27 ^a^	0.698	0.257	<0.001	0.432
C18:3 n-3 (ALA)	1.01 ^a^	1.42 ^a^	0.855 ^a^	1.40 ^a^	0.846 ^a^	1.17 ^a^	0.892 ^a^	1.50 ^a^	0.099	0.214	<0.001	0.403
CLA cis-9,trans-11	0.319 ^a^	0.269 ^a^	0.380 ^a^	0.311 ^a^	0.366 ^a^	0.237 ^a^	0.375 ^a^	0.254 ^a^	0.032	0.555	<0.001	0.318
C20:4 n-6 (ARA)	0.919 ^a^	1.25 ^ab^	0.760 ^a^	1.13 ^ab^	0.714 ^a^	1.05 ^b^	0.916 ^a^	1.41 ^a^	0.090	0.008	<0.001	0.756
C20:5 n-3 (EPA)	0.405 ^a^	0.391 ^a^	0.376 ^a^	0.391 ^a^	0.398 ^a^	0.320 ^a^	0.418 ^a^	0.433 ^a^	0.051	0.722	0.424	0.645
C22:6 n-3 (DHA)	0.378 ^a^	0.172 ^a^	0.156 ^ab^	0.285 ^a^	0.202 ^ab^	0.055 ^a^	0.084 ^b^	0.054 ^a^	0.067	0.013	0.178	0.059
ΣSFA	73.31 ^a^	72.55 ^a^	77.08 ^a^	73.00 ^a^	75.99 ^a^	73.88 ^a^	75.21 ^a^	72.10 ^a^	1.06	0.211	<0.001	0.351
ΣMUFA	15.97 ^a^	14.14 ^a^	14.33 ^a^	14.48 ^a^	15.84 ^a^	13.89 ^a^	15.79 ^a^	13.79 ^a^	0.629	0.853	<0.001	0.083
ΣPUFA	10.72 ^a^	13.31 ^a^	8.59 ^a^	12.52 ^a^	8.17 ^a^	12.23 ^a^	9.00 ^a^	14.11 ^a^	0.810	0.122	<0.001	0.451
Σn-3 PUFA	1.79 ^a^	1.98 ^a^	1.39 ^a^	2.08 ^a^	1.45 ^a^	1.55 ^a^	1.39 ^a^	1.99 ^a^	0.158	0.268	<0.001	0.023
Σn-6 PUFA	8.52 ^a^	11.00 ^a^	6.74 ^a^	10.06 ^a^	6.29 ^a^	10.39 ^a^	7.15 ^a^	11.80 ^a^	0.762	0.185	<0.001	0.461
ΣLC n-3 PUFA	0.782 ^a^	0.563 ^a^	0.531 ^a^	0.677 ^a^	0.600 ^a^	0.375 ^a^	0.503 ^a^	0.486 ^a^	0.096	0.267	0.125	0.052
n-6/n-3	4.89 ^a^	5.30 ^a^	5.02 ^a^	5.02 ^a^	4.71 ^a^	9.30 ^a^	5.24 ^a^	5.99 ^a^	1.32	0.282	0.001	0.020
PUFA/SFA	0.147 ^a^	0.189 ^a^	0.113 ^a^	0.173 ^a^	0.110 ^a^	0.167 ^a^	0.121 ^a^	0.197 ^a^	0.013	0.105	<0.001	0.571

Values are presented as arithmetic means with pooled SEM. C11:0 was used as an internal standard and was excluded before calculation of relative fatty acid proportions; the remaining identified fatty acids were renormalized to 100% in each sample. ΣLC n-3 PUFA = C20:5 n-3 + C22:6 n-3. Data were analyzed using a linear mixed-effects model with maternal dietary treatment, age (day 2 and day 21), and the treatment × age interaction as fixed effects. Dam (ewe) was included as a random intercept, with lamb identity nested within the dam as an additional random intercept to account for clustering of siblings and repeated measurements within the same lamb. All available observations were included in the model, allowing incomplete pairs. Pairwise treatment contrasts within each age and within-treatment age contrasts were adjusted for multiple comparisons using the Holm method. Lowercase superscript letters compare dietary treatments within the same age; means sharing the same letter do not differ significantly (Holm-adjusted *p* ≥ 0.05). Cell n (Day 2/Day 21): Control, 11/12; FO, 15/14; BSFL, 13/13; Mix, 15/14. FO, flaxseed oil; BSFL, black soldier fly larvae oil; Mix, flaxseed oil:BSFL oil blend (2:1); LA, linoleic acid; ALA, α-linolenic acid; ARA, arachidonic acid; EPA, eicosapentaenoic acid; DHA, docosahexaenoic acid; CLA, conjugated linoleic acid; SFA, saturated fatty acids; MUFA, monounsaturated fatty acids; PUFA, polyunsaturated fatty acids; LC-PUFA, long-chain polyunsaturated fatty acids.

**Table 5:** Effects of maternal prepartum lipid supplementation on lamb rearing performance.

Parameter	Treatments				*p*-Value
	Control	Flaxseed Oil	BSFL Oil	Mix ^1^ Oil	
Body weight (day 2) [kg]	3.14 ± 0.25	3.99 ± 0.24	3.46 ± 0.24	3.82 ± 0.24	0.065
Weaning weight (day 56) [kg]	13.65 ± 0.91	14.35 ± 0.89	14.10 ± 0.87	15.90 ± 0.92	0.309
Overall ADG (2–56 d) [g/d]	193.64 ± 14.98	191.61 ± 14.68	195.84 ± 14.18	223.26 ± 15.10	0.377
Survival to Day 56, n/N (%)	17/18 (94.44%)	17/19 (89.47%)	19/20 (95.00%)	17/19 (89.47%)	

^1^ Mix oil consists of flaxseed and BSFL oil in a 2:1 ratio. Data for body weight and average daily gain are presented as least-squares means (LSM) ± model-based standard error (SE). Lamb body weight at day 2, weaning weight at day 56, and average daily gain were analyzed using linear mixed-effects models including maternal dietary treatment, lamb sex, and litter size as fixed effects, with dam included as a random intercept to account for clustering of littermates. Pairwise treatment comparisons were adjusted using the Holm method. Average daily gain (ADG) was calculated for the 54-day period between day 2 and day 56. Survival to Day 56 is presented descriptively as n/N (%), where n is the number of lambs alive at Day 56 and N is the number of live-born lambs included in postnatal follow-up. Sample sizes for body weight measurements (Day 2/Day 56) were: Control, 18/17; FO, 19/17; BSFL, 20/19; Mix, 19/17. For ADG, the corresponding sample sizes were 17, 17, 19, and 17, respectively.

## Data Availability

The datasets used and analyzed during the current study are available from the corresponding author on reasonable request.

## Supplementary Materials

The following supporting information can be downloaded at: https://www.mdpi.com/article/10.3390/ijms27219740/s1.

## Abbreviations

GC–MS	gas chromatography–mass spectrometry
FO	flaxseed oil
BSFL oil	black soldier fly larvae oil
FA	fatty acid
LA	linoleic acid
ALA	α-linolenic acid
ARA	arachidonic acid
DHA	docosahexaenoic acid
CLA	conjugated linoleic acid
EPA	eicosapentaenoic acid
SFA	saturated fatty acids
MUFA	monounsaturated fatty acids
PUFA	polyunsaturated fatty acids
BCFA	branched-chain fatty acids
OBCFA	odd- and branched-chain fatty acids
LC-PUFA	long-chain polyunsaturated fatty acids
SEM	standard error of the mean
FAME	fatty acid methyl esters
ADG	average daily gain
LSM	least-squares means
SD	standard deviation

## References

[1] Khanal P., Nielsen M.O. (2017). Impacts of Prenatal Nutrition on Animal Production and Performance: A Focus on Growth and Metabolic and Endocrine Function in Sheep. J. Anim. Sci. Biotechnol..

[2] Nie Y., Peng X., Li J., Gao Z., Zhang F., Jing W., Liu Y., Nie C. (2026). Appropriate Rumen-Protected Glutamine Supplementation During Late Gestation in Ewes Promotes Lamb Growth and Improves Maternal and Neonatal Metabolic, Immune and Microbiota Functions. Animals.

[3] Wu W., Xu Y., Liu P., Yang Y., Lu H., Rahim M.A., Li B., Liu S., Mao S., Cheng J. (2026). Nutritional Restriction during Late Gestation Disrupts Placental Transcriptional Programs Related to Metabolism, Transport, and Immune Function in a Sheep Model. BMC Genom..

[4] Bell A.W., Hay W.W., Ehrhardt R.A. (1999). Placental Transport of Nutrients and Its Implications for Fetal Growth. J. Reprod. Fertil. Suppl..

[5] Demars J., Fabre S., Sarry J., Rossetti R., Gilbert H., Persani L., Tosser-Klopp G., Mulsant P., Nowak Z., Drobik W. (2013). Genome-Wide Association Studies Identify Two Novel BMP15 Mutations Responsible for an Atypical Hyperprolificacy Phenotype in Sheep. PLoS Genet..

[6] Plante-Dubé M., Létourneau-Montminy M.P., Castonguay F., Gervais R. (2025). Dynamic Responses of Energy Metabolism in Ewes during Late Pregnancy: A Meta-Regression. Animal.

[7] Plante-Dubé M., Bourassa R., Luimes P., Buczinski S., Castonguay F., Gervais R. (2025). Peripartum Energy Metabolism of Prolific Ewes and Their Progeny in Response to Prepartum Feeding and Litter Size. Animal.

[8] van der Linden D.S., Sciascia M., Sales F., McCoard S.A. (2013). Placental Nutrient Transport Is Affected by Pregnancy Rank in Sheep. J. Anim. Sci..

[9] O’Brien K., Wang Y. (2023). The Placenta: A Maternofetal Interface. Annu. Rev. Nutr..

[10] Ander S.E., Diamond M.S., Coyne C.B. (2019). Immune Responses at the Maternal-Fetal Interface. Sci. Immunol..

[11] Ding J., Maxwell A., Adzibolosu N., Hu A., You Y., Liao A., Mor G. (2022). Mechanisms of Immune Regulation by the Placenta: Role of Type I Interferon and Interferon-stimulated Genes Signaling during Pregnancy. Immunol. Rev..

[12] Tanner A.R., Kennedy V.C., Lynch C.S., Hord T.K., Winger Q.A., Rozance P.J., Anthony R.V. (2022). In Vivo Investigation of Ruminant Placenta Function and Physiology—A Review. J. Anim. Sci..

[13] Sammin D., Markey B., Bassett H., Buxton D. (2009). The Ovine Placenta and Placentitis—A Review. Vet. Microbiol..

[14] Tan G.-H., Liu S.-J., Dou M.-L., Zhao D.-F., Zhang A., Li H.-K., Luo F.-N., Shi T., Wang H.-P., Lei J.-Y. (2024). Spatially Resolved Transcriptomic Profiling of Placental Development in Dairy Cow. Zool. Res..

[15] Johnson G.A., Bazer F.W., Seo H., Burghardt R.C., Wu G., Pohler K.G., Cain J.W. (2023). Understanding Placentation in Ruminants: A Review Focusing on Cows and Sheep. Reprod. Fertil. Dev..

[16] Davenport K.M., Ortega M.S., Johnson G.A., Seo H., Spencer T.E. (2023). Review: Implantation and Placentation in Ruminants. Animal.

[17] Edwards A.K., McKnight S.M., Askelson K., McKnight J.R., Dunlap K.A., Satterfield M.C. (2020). Adaptive Responses to Maternal Nutrient Restriction Alter Placental Transport in Ewes. Placenta.

[18] Lang U., Baker R.S., Khoury J., Clark K.E. (2000). Effects of Chronic Reduction in Uterine Blood Flow on Fetal and Placental Growth in the Sheep. Am. J. Physiol. Regul. Integr. Comp. Physiol..

[19] Ma Y., Zhu M.J., Zhang L., Hein S.M., Nathanielsz P.W., Ford S.P. (2010). Maternal Obesity and Overnutrition Alter Fetal Growth Rate and Cotyledonary Vascularity and Angiogenic Factor Expression in the Ewe. Am. J. Physiol. Regul. Integr. Comp. Physiol..

[20] Roque-Jimenez J.A., Oviedo-Ojeda M.F., Whalin M., Lee-Rangel H.A., Relling A.E. (2020). Eicosapentaenoic and Docosahexaenoic Acid Supplementation during Early Gestation Modified Relative Abundance on Placenta and Fetal Liver Tissue MRNA and Concentration Pattern of Fatty Acids in Fetal Liver and Fetal Central Nervous System of Sheep. PLoS ONE.

[21] Omar A., Li Puma L., Risk B., Witt A., Izon C., Whitcomb L., Kareng D., Bouma G., Winger Q., Chicco A. (2022). Impact of Maternal Omega-3 Fatty Acid Intake on Ovine Placental and Fetal Tissue Metabolism. Curr. Dev. Nutr..

[22] Noble R.C., Shand J.H., Calvert D.T. (1982). The Role of the Placenta in the Supply of Essential Fatty Acids to the Fetal Sheep: Studies of Lipid Compositions at Term. Placenta.

[23] Steinhauser C.B., Askelson K., Lambo C.A., Hobbs K.C., Bazer F.W., Satterfield M.C. (2021). Lipid Metabolism Is Altered in Maternal, Placental, and Fetal Tissues of Ewes with Small for Gestational Age Fetuses. Biol. Reprod..

[24] Elmes M., Tew P., Cheng Z., Kirkup S.E., Abayasekara D.R.E., Calder P.C., Hanson M.A., Wathes D.C., Burdge G.C. (2004). The Effect of Dietary Supplementation with Linoleic Acid to Late Gestation Ewes on the Fatty Acid Composition of Maternal and Fetal Plasma and Tissues and the Synthetic Capacity of the Placenta for 2-Series Prostaglandins. Biochim. ET Biophys. Acta (BBA)—Mol. Cell Biol. Lipids.

[25] Coleman D.N., Murphy K.D., Relling A.E. (2018). Prepartum Fatty Acid Supplementation in Sheep. II. Supplementation of Eicosapentaenoic Acid and Docosahexaenoic Acid during Late Gestation Alters the Fatty Acid Profile of Plasma, Colostrum, Milk and Adipose Tissue, and Increases Lipogenic Gene Expression of Adipose Tissue. J. Anim. Sci..

[26] Carranza-Martin A.C., Nickles K., Sherlock D.N., Relling A.E. (2025). Supplementation with Eicosapentaenoic and Docosahexaenoic Acids during Late Gestation Alters Fatty Acid Profiles in Ewe Colostrum, Milk, and Plasma, and Lamb Plasma. J. Anim. Sci..

[27] Lewandowska K., Pachura-Hanusek N., Burek A., Szumny A., Kupczyński R. (2026). Low n-3/High n-6 Flaxseed and Hermetia illucens Larvae Oils as Feed Materials: Effects on In Vitro Rumen Fermentation, Maternal Metabolism, Antioxidant Status and Colostrum in Prolific Ewes. Int. J. Mol. Sci..

[28] Hervás G., Toral P.G., Labbouz Y., Baila C., Boussalia Y., Frutos P. (2025). Replacing Soybean Meal with House Cricket (*Acheta domesticus*) Meal in Ruminant Diet: Effects on Ruminal Fermentation, Degradation, and Biohydrogenation. J. Insects Food Feed..

[29] Baila C., Frutos P., Renna M., Barrio E., Badino P., Trespeuch C., Gasco L., Hervás G., Toral P.G. (2026). Replacing Palm Fatty Acid Distillate by Black Soldier Fly Oil in the Diet of Dairy Ewes: Effects on Animal Performance, Ruminal Fermentation, Plasma Metabolites, and Oxidative Stress. J. Dairy Sci..

[30] Bogolyubova N.V., Devyatkin V.A., Nekrasov R.V. (2025). Rumen Metabolism and Gas Formation in Sheep Fed Different Fat Sources. Agric. Sci. Euro-North-East.

[31] Lewandowska K., Pachura-Hanusek N., Szumny A., Zielak-Steciwko A., Kupczyński R. (2026). Effects of Prepartum Supplementation with Flaxseed and Black Soldier Fly Larvae Oils on Fatty Acid Composition and Lipid Quality Indices in Sheep Milk. Molecules.

[32] (2010). European Union Directive 2010/63/EU of the European Parliament and of the Council of 22 September 2010 on the Protection of Animals Used for Scientific Purposes. Off. J. Eur. Union.

