# Supplementary material for: Effects of Prepartum Flaxseed and Hermetia illucens Oil Supplementation on Fatty Acid Profiles Across the Maternal–Placental–Offspring Axis in Prolific Ewes
Source: Int J Mol Sci. 2026 Oct 30;27(21):9740. doi: 10.3390/ijms27219740 (PMC13762940; doi:10.3390/ijms27219740)
Supplement: Supplementary file 1 [file ijms-27-09740-s001.zip › ijms-4615857-supplementary.pdf]

Supplementary Table S1. Benjamini–Hochberg false discovery rate adjustment of fatty acid endpoints and derived indices.

Unadjusted omnibus *p*-values and FDR-adjusted *q*-values are presented to three decimal places; values below 0.001 are reported as <0.001. Adjustments were performed separately for each model term within each biological compartment. Bold *q*-values indicate FDR significance (q < 0.05), determined using unrounded values.

Panel A. Maternal serum

Fatty acid / index	Treatment		Time		Treatment × time	
	p	*q*	*p*	*q*	*p*	*q*
C14:0	<0.001	**<0.001**	<0.001	**<0.001**	<0.001	**<0.001**
C14:1	0.013	0.146	<0.001	**<0.001**	0.003	**0.023**
anteiso-C15:0	0.410	0.619	0.005	**0.009**	0.154	0.394
C15:0	0.055	0.265	<0.001	**<0.001**	<0.001	**<0.001**
iso-C16:0	0.403	0.619	<0.001	**<0.001**	0.013	0.057
C16:0	0.109	0.412	0.034	0.056	0.994	0.994
anteiso-C16:0	0.381	0.619	0.907	0.919	0.647	0.879
C16:1	0.026	0.177	0.150	0.204	0.876	0.960
anteiso-C17:0	0.419	0.619	0.243	0.318	0.485	0.749
C17:0	0.094	0.400	<0.001	**<0.001**	0.517	0.764
C17:1	0.692	0.774	0.851	0.905	0.245	0.538
iso-C18:0	0.578	0.701	<0.001	**<0.001**	0.156	0.394
C18:0	0.210	0.539	<0.001	**<0.001**	0.740	0.879
C18:1 cis-9	0.254	0.539	0.640	0.725	0.670	0.879
C18:2 trans	<0.001	**0.008**	<0.001	**<0.001**	<0.001	**<0.001**
C18:2 cis n-6	0.573	0.701	0.006	**0.011**	0.693	0.879
C18:3 n-6	0.232	0.539	0.088	0.124	0.372	0.653
C18:3 n-3	0.232	0.539	<0.001	**<0.001**	0.001	**0.009**
C20:0	0.219	0.539	0.608	0.712	0.011	0.057
C20:3 n-6	0.250	0.539	0.008	**0.014**	0.013	0.057
C20:4 n-6 — ARA	0.023	0.177	<0.001	**<0.001**	0.162	0.394
C22:0	0.729	0.774	0.830	0.905	0.365	0.653
C20:5 n-3 — EPA	0.714	0.774	<0.001	**<0.001**	0.849	0.960
C22:6 n-3 — DHA	0.674	0.774	<0.001	**<0.001**	0.253	0.538
SFA	0.955	0.955	0.919	0.919	0.949	0.978
MUFA	0.050	0.265	0.365	0.460	0.937	0.978
PUFA	0.826	0.851	0.461	0.559	0.709	0.879
n-6 PUFA	0.469	0.638	0.037	0.058	0.750	0.879
n-3 PUFA	0.559	0.701	<0.001	**<0.001**	0.024	0.090
LC-PUFA	0.406	0.619	<0.001	**<0.001**	0.384	0.653
BCFA	0.455	0.638	0.045	0.067	0.363	0.653
OCFA	0.124	0.423	<0.001	**<0.001**	0.153	0.394
OBCFA	0.382	0.619	<0.001	**<0.001**	0.152	0.394
n-6/n-3	0.332	0.619	<0.001	**<0.001**	0.463	0.749

Note. Repeated-measures model: dietary treatment, time, and treatment × time. Post hoc procedures are described in the main manuscript.

Panel B. Placental tissue

Fatty acid / index	Treatment p	Treatment q (BH)
C14:0	0.649	0.881
iso-C15:0	0.017	0.516
anteiso-C15:0	0.575	0.875
C15:0	0.834	0.942
iso-C16:0	0.200	0.611
C16:0	0.955	0.983
anteiso-C16:0	0.820	0.942
C16:1	0.436	0.847
anteiso-C17:0	0.426	0.847
C17:1	0.227	0.611
C18:0	0.932	0.983
C18:1 cis-9	0.533	0.875
C18:1 isomer	0.828	0.942
C18:2 cis n-6	0.395	0.847
C18:3 n-6	0.654	0.881
C20:0	0.618	0.881
C20:1	0.542	0.875
C20:2	0.531	0.875
C21:0	0.697	0.904
C20:3 n-6	0.148	0.611
C20:4 n-6 — ARA	0.269	0.672
C22:0	0.872	0.953
C22:1	0.152	0.611
C24:1	0.382	0.847
C22:6 n-3 — DHA	0.213	0.611
SFA	0.990	0.990
MUFA	0.559	0.875
PUFA	0.030	0.516
BCFA	0.086	0.611
OCFA	0.753	0.942
OBCFA	0.047	0.547
n-6 PUFA	0.158	0.611
n-3 PUFA	0.213	0.611
LC-PUFA	0.138	0.611
n-6/n-3	0.162	0.611

Note. Group comparisons: omnibus dietary treatment effect. Post hoc procedures are described in the main manuscript.

Panel C. Amniotic fluid

Fatty acid / index	Treatment p	Treatment q (BH)
C12:0	0.857	0.897
C14:0	0.728	0.801
C16:0	0.132	0.362
C16:1	0.666	0.771
C17:1	0.054	0.299
C18:0	0.032	0.272
C18:1 trans-9	0.210	0.463
C18:2 cis n-6	0.262	0.472
C20:0	0.037	0.272
C20:1	0.005	0.109
C21:0	0.103	0.324
C20:3 n-6	0.417	0.573
C20:3 n-3	0.550	0.672
C22:0	0.337	0.495
SFA	0.167	0.409
MUFA	0.081	0.324
PUFA	0.301	0.472
OCFA	0.103	0.324
n-6 PUFA	0.279	0.472
n-3 PUFA	0.550	0.672
LC-PUFA	0.960	0.960
n-6/n-3	0.268	0.472

Note. Group comparisons: omnibus dietary treatment effect. Post hoc procedures are described in the main manuscript.

Panel D. Lamb serum

Fatty acid / index	Treatment		Age		Treatment × age	
	p	*q*	*p*	*q*	*p*	*q*
C10:0	0.236	0.634	0.046	0.071	0.023	0.156
C12:0	0.017	0.129	0.461	0.545	0.053	0.224
iso-C14:0	0.924	0.990	<0.001	**<0.001**	0.387	0.625
anteiso-C14:0	0.791	0.990	<0.001	**<0.001**	0.116	0.348
C14:0	0.079	0.511	0.002	**0.004**	0.333	0.608
iso-C15:0	0.968	0.991	<0.001	**<0.001**	0.885	0.923
C14:1 cis-9	0.906	0.990	<0.001	**<0.001**	0.856	0.917
C15:0	0.969	0.991	0.108	0.152	0.348	0.608
C15:1 cis-10	0.908	0.990	<0.001	**<0.001**	0.756	0.873
iso-C16:0	0.856	0.990	<0.001	**<0.001**	0.392	0.625
C16:0	0.004	0.080	<0.001	**<0.001**	0.721	0.873
C16:1 trans-9	0.834	0.990	0.226	0.291	0.163	0.446
C16:1 cis-7	0.104	0.523	0.068	0.098	0.199	0.471
iso-C17:0	0.775	0.990	0.054	0.081	0.251	0.537
anteiso-C17:0	0.797	0.990	0.638	0.701	0.179	0.447
C17:0	0.540	0.990	0.816	0.835	0.007	0.156
C17:1 cis-10	0.774	0.990	0.032	0.051	0.005	0.156
C18:0	0.005	0.080	<0.001	**<0.001**	0.946	0.946
C18:1 trans-11	0.005	0.080	0.023	**0.038**	0.224	0.505
C18:1 cis-9	0.858	0.990	<0.001	**<0.001**	0.100	0.320
C18:1 cis-11	0.296	0.634	<0.001	**<0.001**	0.060	0.224
C18:1 cis-12	0.321	0.656	0.009	**0.015**	0.025	0.156
C18:2 trans,cis	0.453	0.886	<0.001	**<0.001**	0.903	0.923
C18:2 cis-9,cis-12	0.257	0.634	<0.001	**<0.001**	0.432	0.629
C20:0	0.733	0.990	0.755	0.791	0.811	0.890
C18:3 n-3	0.214	0.634	<0.001	**<0.001**	0.403	0.625
CLA cis-9,trans-11	0.555	0.990	<0.001	**<0.001**	0.318	0.608
C20:3 n-6	0.713	0.990	0.728	0.780	0.792	0.890
C22:0	0.610	0.990	0.588	0.678	0.607	0.780
C20:4 n-6	0.008	0.087	<0.001	**<0.001**	0.756	0.873
C20:5 n-3	0.722	0.990	0.424	0.520	0.645	0.806
C22:6 n-3 — DHA	0.013	0.115	0.178	0.235	0.059	0.224
ΣSFA	0.211	0.634	<0.001	**<0.001**	0.351	0.608
ΣMUFA	0.853	0.990	<0.001	**<0.001**	0.083	0.287
ΣPUFA	0.122	0.551	<0.001	**<0.001**	0.451	0.629
ΣUFA	0.211	0.634	<0.001	**<0.001**	0.351	0.608
Σn-3 PUFA	0.268	0.634	<0.001	**<0.001**	0.023	0.156
Σn-6 PUFA	0.185	0.634	<0.001	**<0.001**	0.461	0.629
ΣLC n-3 PUFA (EPA+DHA)	0.267	0.634	0.125	0.171	0.052	0.224
ΣBCFA	0.991	0.991	0.973	0.973	0.169	0.446
ΣOCFA	0.677	0.990	0.428	0.520	0.017	0.156
ΣOBCFA	0.881	0.990	0.629	0.701	0.028	0.156
n-6/n-3	0.282	0.634	0.001	**0.002**	0.020	0.156
PUFA/SFA	0.105	0.523	<0.001	**<0.001**	0.571	0.755
UFA/SFA	0.179	0.634	<0.001	**<0.001**	0.435	0.629

Note. Mixed-effects model: dietary treatment, age, and treatment × age. Post hoc procedures are described in the main manuscript.
